# Settlement and Growth of *Mytilus galloprovincialis* Pediveliger Larvae in Response to Biofilm-Based Microalgae and Chemical Neuroactive Compounds

**DOI:** 10.3390/biology15010010

**Published:** 2025-12-19

**Authors:** Hafsa Janah, Yassine Ouagajjou, Adil Aghzar, Pablo Presa

**Affiliations:** 1 Research Team of Agriculture and Aquaculture Engineering (G2A), Polydisciplinary Faculty of Larache, Ab-Delmalek Essaadi University, Tetouan 93000, Morocco; hafsa.janah@etu.uae.ac.ma (H.J.); y.ouagajjou@uae.ac.ma (Y.O.); a.aghzar@uae.ac.ma (A.A.); 2 Amsa Shellfish Research Station, National Institute of Fisheries Research, Tetouan 93000, Morocco; 3 Laboratory of Marine Genetic Resources (ReXenMar), CIM—Universidade de Vigo, 36310 Vigo, Spain

**Keywords:** *Mytilus galloprovincialis*, mussel aquaculture, biofilm, chemical inducers, GABA, KCl, metamorphosis, larvae settlement, hatchery production

## Abstract

The sustainability of mollusc aquaculture relies, in part, on overcoming the challenges of spat production in captivity, particularly during the metamorphosis and settlement stages. The optimization of rearing technologies at these stages would ensure possible solutions for sustainably producing mollusc spat while simultaneously improving stock performance. The current work represents a large-scale trial examining the effect of biological and chemical inducers on larval settlement in *Mytilus galloprovincialis*. For this purpose, one batch of pediveliger larvae was directly transferred to settlement on microalgae-based biofilm (mature cylinders), while another batch was pretreated with gamma-aminobutyric acid GABA (10^−4^ M, 10^−5^ M and 10^−6^ M) and potassium chloride KCl (20 mM and 30 mM) according to two different exposure times (6 h and 24 h), before being transferred for settlement (immature cylinders). The impact of different treatments on larval performance was evaluated in terms of larval settlement rate (*Sr*), post-larval growth rate (*Gr*), and spat production rate (*Pr*). The biofilm treatment had the highest settlement rate and spat production (*Sr* = 65% and *Pr* = 46.4 spat/cm^2^) compared to chemical treatments. The highest settlement rate among chemical treatments occurred under short exposure times (6 h) to low GABA concentrations, i.e., *Sr* 40% and 45% at GABA 10^−5^ M and 10^−6^ M, respectively). GABA and KCl treatments ensured a faster post-larval growth rate than the biofilm, i.e., 15.54 ± 7.67 µm/day, 18.26 ± 9.39 µm/day, and 11.35 ± 6.73 µm/day, respectively, while control trials showed the lowest growth rate (6.80 ± 4.39 µm/day). These findings reveal a key trade-off: biofilm is the most effective measure for promoting spat production, while a targeted use of GABA and KCl at short exposure times (6 h) appears to significantly enhance post-larvae growth.

## Simple Summary

Technical procedures and breeding systems need to be optimized to ensure the economic viability and sustainability of farm-raised mussel production. The blue mussel *Mytilus galloprovincialis* plays a crucial economic and environmental role, necessitating improvements in breeding due to the scarcity of natural seed sources. The present study aimed to evaluate the efficiency of biofilm in conjunction with chemical inducers (GABA and KCl) on larval settlement, post-larval growth, and spat production in a large-scale operational trial. This research offers a standardized approach to accelerate the production cycle and maximize its results, while maintaining optimal seed quality.

## 1. Introduction

Aquaculture has experienced historic growth, surpassing capture fisheries for the first time in 2022, with finfish, crustaceans, and molluscs driving this expansion [1]. Mollusc spat production underpins aquaculture sustainability by ensuring a reliable, seasonal, and demand-based supply [2]. Particularly, bivalves are crucial ecosystem engineers, creating habitats that provide food and shelter for diverse groups of taxa [3]. Despite their ecological and economic importance, mollusc production often depends on seed resources collected from the marine intertidal zone [4]. However, wild mollusc populations exhibit significant annual fluctuations because their biomass restitution depends on the success of natural reproduction [5]. The spatial and temporal variability of seed abundance is directly influenced by anthropogenic stressors. For instance, eutrophication and mercury pollution released from urban areas have decreased the diversity of mollusc assemblages in the Caribbean Sea [6]. Similarly, habitat alteration has obstructed pearl oyster recruitment, as reported in the mid-Atlantic region, due to commercial dredging boats [7]. Also, overfishing counts as a primary driver in the decline of bivalve populations by reducing their reproductive success [8]. Climate change is an additional growing threat, with bivalve populations projected to be at significant risk from heat waves, which are expected to triple by 2040 [9]. Namely, ocean warming in the northern hemisphere has been linked to declines in the nutritional quality of bivalves, i.e., in levels of lipids and carbohydrates [10]. High mortality of the grooved carpet shell *Ruditapes decussatus* juveniles due to rising temperatures was also reported in coastal areas of Portugal (Ria Formosa lagoon) [11]. Likewise, increasingly frequent harmful algal blooms (HABs) have been reported to threaten bivalve spat survival and harvests in the Mediterranean Sea [12].

It is well known that mussel populations play a vital role as filter feeders, increasing water clarity by reducing algal blooms and other particulate matter [13]. In extractive aquaculture, this filtration function is leveraged to optimize nutrient removal from coastal ecosystems. However, challenges such as depletion of natural seed stocks hinder the sustainability of this activity. Meanwhile, commercial mussel aquaculture for human consumption is continually optimizing all stages of production to address seed shortages and meet market demands [14]. These optimization efforts include genetic management and selection [15,16], broodstock management [17], spawning techniques [18], larval rearing protocols [19,20,21], and post-larval metamorphosis and settlement [22,23]. The latter are of primary concern in bivalve aquaculture [24] because their unpredictable success rate often determines production efficiency [25]. Despite the recent discovery of several morphogenetic inducers, the molecular processes underlying the development of larval competence and the induction of metamorphosis in marine molluscs remain poorly understood [26].

The widely distributed mussel *Mytilus galloprovincialis* [27] is a species of great global value due to its economic importance [28] and ecological contribution [29]. However, overreliance on natural beds for seed supply has led to uneven recruitment of seed stocks, creating a bottleneck for sustainable production in recent decades [30,31]. Similar to other marine invertebrates, this species undergoes a metamorphic transition, moving from a pelagic (free-swimming) larval stage to a substrate-attached sessile stage. This natural process is modulated by exogenous cues of diverse physical and chemical origins, such as surface texture and water flow in the greenshell mussel *Perna canaliculus* [32,33], bacterial biofilms in *Mytilus coruscus* [34], pharmacological compounds in *Mytilus edulis* [35], bacterial compounds in *Mytilopsis sallei* [36], or macroalgal induction in *M. galloprovincialis* [22]. Therefore, chemical inducers have arisen in interest due to their ability to mimic the activity of naturally occurring inducers. A putative settlement inducer is the accessory photosynthetic pigment of coralline red algae, which contains two cyclic imino analogs of γ-aminobutyric acid (GABA) [37]. This neurotransmitter has successfully induced settlement in the abalone *Haliotis discus hannai* [38] and in *M. galloprovincialis* [24]. GABA is a non-proteinogenic amino acid that can be naturally produced by marine cyanobacteria [39], bacteria [40], and yeast [41]. The influence of GABA on mollusc larvae settlement and viability has been shown by reducing ciliary beating and hindering larvae’s swimming behavior, e.g., GABA blocked serotonin’s excitatory effect on gill lateral cilia beating in the oyster *Crassostrea virginica* [42]. Additionally, GABA may affect the immune system by suppressing the phagocytic activity of hemocytes (immune cells), as observed in the blood clam *Tegillarca granosa* [43]. Such phagocytosis is a key defense mechanism in mussel larvae, which initiates as early as 24 h post-fertilization and increases during the metamorphosis stage due to environmental cues [44].

Similarly, potassium chloride (KCl) has been shown to be effective in inducing settlement in the marine bryozoan *Bugula neritina* [45] as well as in the oyster *Pinctada fucata martensii* [46]. Its induction is thought to occur through a rise in extracellular K+ ions, which leads to the depolarization of excitable cells, ultimately triggering metamorphic pathways [47,48]. The role of K+ ions on diverse metabolic pathways in aquatic species is controversial, with some studies reporting negative effects on growth and others showing a positive influence on larval metamorphosis and development [49,50]. In addition, the effect of salinity on digestive enzyme activity and nutrient absorption has been shown to influence growth in other aquatic species [50,51,52,53]. Beyond inorganic molecules, compounds of organic origin, such as microalgae, have shown their potential as settlement inducers in the black-footed Abalone *Haliotis iris* [54] or in the soft-coral larvae of *Alcyonium siderium Verrill* [55]. This inductive property can be attributed to the production of compounds with antimicrobial properties as well as to the promotion of beneficial microorganisms that can be ingested by larvae and enhance their survival and performance [56,57].

Provided that a significant enhancement in settlement efficiency would allow us to optimize the mass production of mussels in a hatchery, in this work, we aimed to test the effect of putative inducers of larvae performance in *M. galloprovincialis*. Specifically, we tested the effect of chemical (γ-aminobutyric acid, GABA, and potassium chloride, KCl) as well as biological (microalgal biofilm) treatments on larval settlement, post-larval growth rate, and spat production. The working hypothesis was that the putative chemical or biological inducers would not produce a differential effect on those physiological and behavioral processes compared to the control trial.

## 2. Materials and Methods

### 2.1. Spawning and Larval Culture

**Figure 1:**
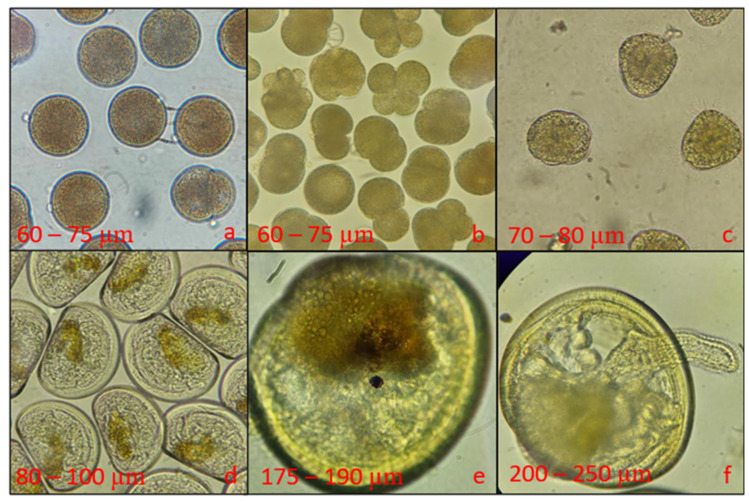
Major development stages of *M. galloprovincialis* larvae. (**a**) Appearance of second polar body upon fertilization, (**b**) egg divisions, (**c**) trochophore larvae, (**d**) D-shape larvae, (**e**) eyespot larvae, and (**f**) pediveliger larvae.

**Figure 2:**
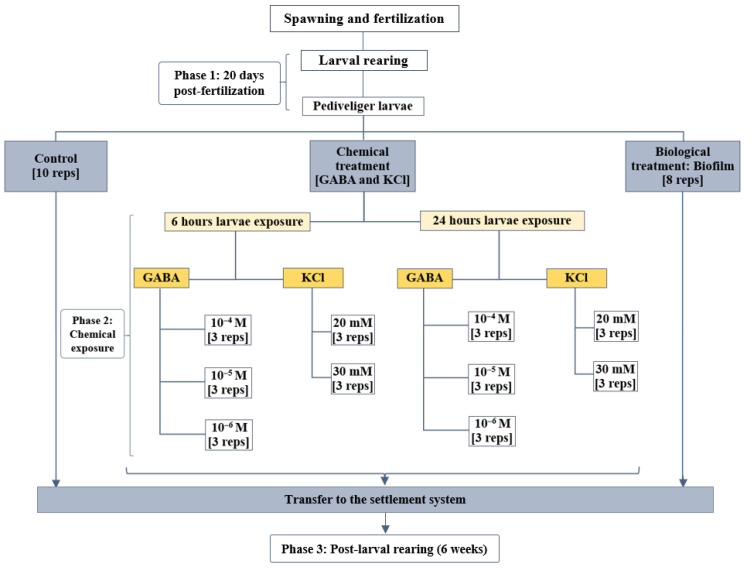
Experimental design to test the effect of chemical (GABA; KCl) and biofilm treatments on *M. galloprovincialis* larval settlement; reps, No. of replicates per treatment.

In January 2023, 40 adult *M. galloprovincialis* mussels were collected from a longline rearing structure in AMSA Bay (35°31′59.5″ N 5°13′29.0″ W) and brought to the Amsa Shellfish Research Station to conduct the current experiment. All the specimens were meticulously cleaned and arranged in two 10 L polycarbonate basins (Sanko Co., Ltd., Tokyo, Japan). The spawning protocol consisted of thermal stimulation by alternating the specimens between cold (14 ± 1.5 °C) and hot (26 ± 1 °C) water baths for multiple cycles. Once spawning responses took place, each individual was transferred to a separate beaker with filtered and UV-treated seawater (FSW) to spawn individually and to avoid gamete inter-contamination. Oocytes were sieved through a 20 µm mesh and assessed for quality (roundness) and quantity using a profile projector (V-12B, Nikon Corp., Tokyo, Japan). The density of spermatozoids was evaluated using a Malassez counting chamber (model BR719005-1EA, Brand GmbH + Co KG, Wertheim, Germany). Oocytes were carefully placed in a 200 L polycarbonate tank (Sanko Co., Ltd., Tokyo, Japan) and fertilized at a ratio of 150 spermatozoids per oocyte. After two hours of incubation, fertilized eggs were sieved through a mesh net (20 µm) and washed thoroughly with FSW to remove excess sperm. The number of eggs successfully fertilized was averaged from three subsamples (25 μL), as counted using a profile projector (Nikon V-12B). Fertilized oocytes were transferred to a 1000 L tank (Sanko Co., Ltd., Tokyo, Japan) with FSW at 20 °C at an initial density of 20 eggs/mL. After 48 h, straight-hinge veliger larvae appeared, and their rearing consisted of keeping them at 20.1 ± 0.2 °C, with water change and sieving every 48 h until the 20th dpf (day post-fertilization). Larvae cultures were fed a diet of *Isochrysis galbana* and *Chaetoceros gracilis* (15–40 cells/µL each) daily, and that quantity was increased with rearing age (Figure 1). Larvae that reached pediveliger stage (≥250 µm in shell length) were transferred to the settlement system, i.e., phase 1 in Figure 2.

### 2.2. Preparation of Biofilm and Chemical Inducers

#### 2.2.1. Microalgae Biofilm

Microalgae strains *Chaetoceros gracilis* (0.5–0.7 × 10^6^ cell/mL), *Pavlova lutheri* (1–2 × 10^6^ cell/mL), and *Tetraselmis suecica* (0.3–0.4 × 10^6^ cell/mL) cultured at the phytoplankton production unit of the AMSA hatchery were used to prepare the biofilm surface. The inoculum composition consisted of the diatom *C. gracilis* (ranging 21–27%) and the flagellates *P. lutheri* (ranging 55.6–60.7%) and *T. suecica* (ranging 16.7–18.2%). Eight cylinders (150 µm mesh (NBK Co., Nagoya, Japan), porosity, 300 mm diameter) were immersed in a 150 L mixture of those microalgae cultures in a rectangular tank. The tank was emptied twice a week, and microalgae cultures were renewed for three weeks. The nylon mesh cylinders were kept without any cleaning until the biofilm formed. The maturity of the biofilm was monitored primarily by visual confirmation and microscopic observation during the three-week growth period. Biochemical quantification of the attached biomass was not performed in this operational trial. The cylinders were then transferred to the settlement system described in Section 2.3.

#### 2.2.2. Preparation of GABA and KCl Solutions

The stock solution of γ-aminobutyric acid (GABA, ≥99%, Sigma-Aldrich, St. Louis, MO, USA) was prepared prior to the bioassays by dissolving 1.742 g of GABA powder in 100 mL of distilled water to prepare a 1 M stock solution. This stock solution was used to prepare 10 L of filtered and UV-treated seawater (FSW) at serial concentrations of 10^−4^ M, 10^−5^ M, and 10^−6^ M, successively. Potassium chloride (KCl, analytical grade, Merck KGaA, Darmstadt, Germany) solutions were prepared by dissolving 14.91 g and 22.37 g in 10 L of FSW to achieve 20 mM and 30 mM KCl solutions, respectively.

### 2.3. Experimental Assays

#### 2.3.1. Settlement System Set-Up

**Figure 3:**
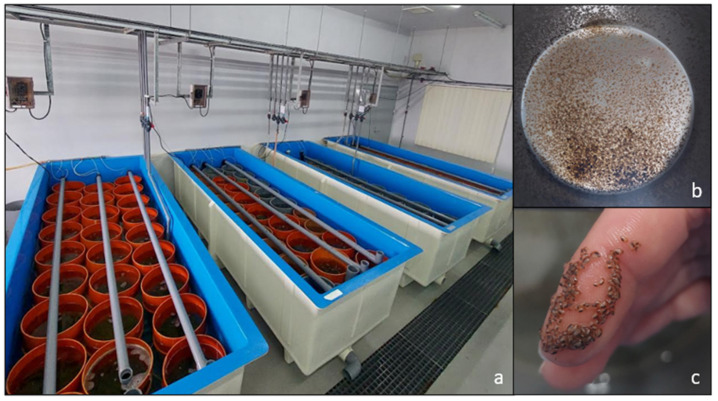
(**a**) Micro-nursery system with the cylinders used in the settlement phase, (**b**) spat settled on a mesh-bottomed cylinder, and (**c**) close-up photo of mussel spat after 25 days of rearing in the settlement system.

The experimental period lasted nine weeks and was divided into three phases: phase 1: larval pre-exposure, phase 2: larvae’s exposure to treatments, and phase 3: post-larval rearing (Figure 2). Phase 1 lasted 20 days, i.e., until larvae reached the pediveliger stage. The total number of larvae (2 × 10^6^) was estimated as the average number of three 25 μL subsamples counted using a profile projector (Nikon V-12B). In phase 2, pediveliger larvae designated as the control group and the biofilm group were distributed directly into the settlement system at a density of 70 larvae/cm^2^ (50,000 larvae per cylinder), while larvae destined for chemical treatments were exposed to various concentrations of GABA (10^−4^ M, 10^−5^ M, and 10^−6^ M) and KCl (20 mM and 30 mM). Phase 2 was enforced for 6 h and for 24 h, and then larvae were transferred to the settlement system for post-larval rearing. In phase 3, post-larvae were kept in the settlement system for 6 weeks and comprised 48 cylinders (including replicates) distributed as follows: control group (10 replicates), biofilm group (8 replicates), and 3 replicates per chemical treatment under both, 6 h and 24 h exposure to the three GABA concentrations and the two KCl concentrations (Figure 2). The settlement system consisted of a 1200 L rectangular fiberglass tank (Sanko Co., Ltd., Tokyo, Japan) with a series of cylinders (300 mm in diameter) bottomed with a 150 µm nylon mesh as previously described [58]. Throughout the experimental period, post-larvae were maintained in the settlement system with constant aeration and water downwelling flowthrough (Figure 3a). Routinely, the following physicochemical characteristics were currently measured: temperature (20.18 ± 0.49 °C), dissolved oxygen (7.01 ± 0.02 mg/L), salinity (37.14 ± 0.41 psu), and pH (8.74 ± 0.06).

#### 2.3.2. Feeding and Maintenance During Settlement

During settlement, larvae were fed a multi-algal diet of three strains (20,000 cells/mL of *T. suecica*, 50,000 cells/mL of *I. galbana*, 100,000 cells/mL of *C. calcitrans*) daily. Regularly (every 48 h), settlement systems (cylinders) were carefully taken out of the tank and cleaned with filtered seawater to rid them of any debris, microalgae residues, and dead larvae.

#### 2.3.3. Assessment of Larval Settlement, Post-Larval Growth Rate, and Spat Production

Larvae settlement rate (*Sr*) and spat production (*Pr*) were estimated at the end of the experiment using the following formulas [58]. The settlement rate (*Sr*) was defined as the percentage of post-larvae settled on the nylon mesh cylinder and was estimated per cylinder using the following formula:

(1) Sr (%) = (Nf/Ni) × 100

where *Sr* is the settlement rate; *Nf* is the final number of produced post-larvae; *Ni* is the initial number of seeded larvae.

At the end of the experiment (phase 3), the settled post-larvae from each nylon mesh cylinder were collected and weighed, and the final number of post-larvae (*Nf*) was calculated using the following formula:

(2) $$Nf\;=\;(Wt/\bar{W}i)$$

where *Wt* is the total weight of settled post-larvae per replicate, and $\bar{W}i$ is the individual average weight per post-larvae as estimated using a random subsample and the following expression:

(3) $$\bar{W}i\;=\;(Wsub/Nsub)$$

where *Wsub* $=\;\sum_{j=1}^{N_{sub}}wj$ is the total weight of the subsample per replicate (with w*j* representing the weight of the *j*th post-larvae in the subsample), and *Nsub* is the total number of post-larvae per subsample and replicate.

The spat production (*Pr*) per cm^2^ was calculated as follows:

(4) Pr = (Nf/S)

where *Pr* is the spat production (spat/cm^2^), *Nf* is the final number of settled post-larvae, and *S* is the surface area of the cylinder mesh (cm^2^).

In order to evaluate the post-larval growth rate during the first week in the settlement system, ~20 post-larvae were sampled from each cylinder for biometric measurement (length and width) using the profile projector (Nikon V-12B). Growth rate (*Gr*) was then calculated per treatment using the following formula:

(5) $$Gr=\left({\bar{M}}_{1}-\;{\bar{M}}_{0}\right)/t$$

where *Gr* is the growth rate (µm/day); ${\bar{M}}_{0}$ is the initial average length of pediveliger larvae, ${\bar{M}}_{1}$ is the final average length of post-larvae, and *t* is the number of days elapsed since the day of transfer of pediveliger larvae to the settlement system (one week of post-larval rearing).

### 2.4. Data Analysis

One-way ANOVA analyses (Fisher test) were conducted to determine the effect of treatments on larvae settlement, post-larval growth rate, and spat production. Tests’ significance was assessed at an alpha threshold of 0.05, as corrected with the Welch test [59]. Boxplots were elaborated to visualize settlement rates and spat production per treatment. Heatmaps were developed to represent Tukey test results between treatments. Prior to univariate analysis, data was transformed when necessary to meet the assumptions of normality and homogeneity of variance. Principal Component Analysis (PCA) and Redundancy Analysis (RDA) were applied to combinations of larval settlement, spat production, and post-larval growth in response to the different treatments. For the multivariate analyses, all variables were standardized prior to analysis. All the statistical analyses were conducted in R software (version 4.1.0, R Foundation for Statistical Computing, Vienna, Austria, 29 May 2021), the Rcmdr package (version 2.7.2), and the RStudio integrated development environment (version 2022.12.0, Posit Software, PBC, Boston, MA, USA).

## 3. Results

### 3.1. Effect of Treatments on Larvae Settlement and Spat Production at Different Exposure Times

**Figure 4:**
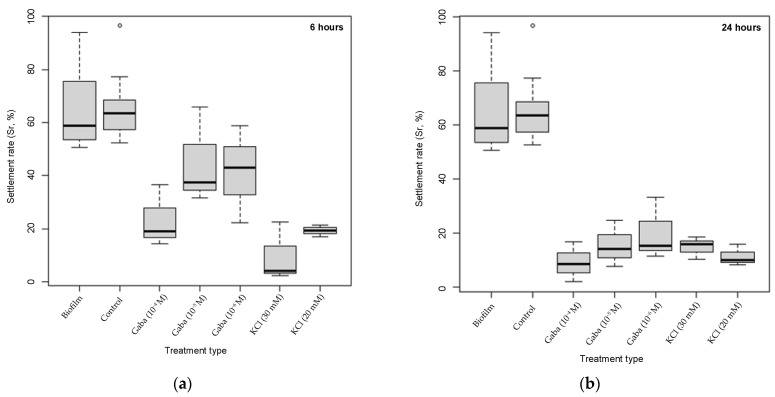
*M. galloprovincialis* larvae settlement rate (*Sr*, %) under chemical treatments (GABA, KCl), biofilm treatment, and control group. (**a**) Chemical exposure for 6 h, and (**b**) chemical exposure for 24 h. The open circle represents an outlier value.

The settlement rate of larvae (*Sr*) after 6 h was higher in the biofilm treatment (65%) and in the control group (64.9%) than in GABA and KCl treatments (Figure 4). This difference was accentuated after 24 h. A negative correlation was observed between *Sr* and chemical treatments at both exposure times, except under KCl after 24 h, where that correlation was barely positive.

The ANOVA test showed that exposure time of larvae to GABA negatively affects their settlement (*F* = 10.73; *df*_num = 1; *df*_den = 12.26; *p* < 0.01). In contrast, exposure time to KCl had no effect on *Sr* (*F* = 0.11; *df*_num = 1; *df*_den = 7.06; *p* > 0.05) (Table 1).

**Figure 5:**
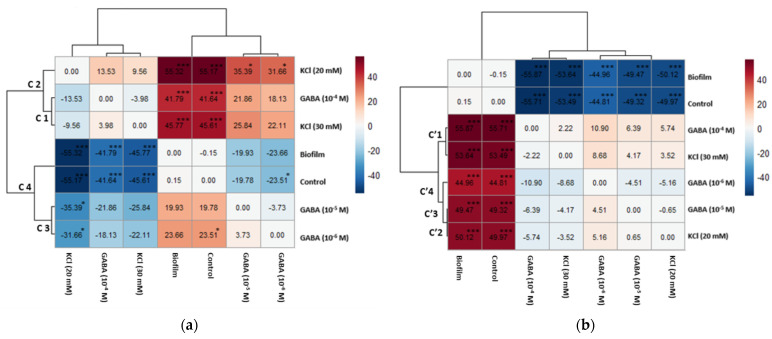
Heatmaps of Tukey HSD pairwise results for larval settlement rates (*Sr*, %) for biofilm, GABA, KCl, and control (**a**) after 6 h exposure to chemical treatments (hierarchical dendrogram Cn), and (**b**) after 24 h exposure to chemical treatments (hierarchical dendrogram C′n). Upper diagonal cells contain the mean difference in settlement rates (*Sr*, %) between the ‘Compared Treatment’ (column) and the ‘Reference Treatment’ (row). Lower diagonal shows (Mean of Row Treatment)–(Mean of Column Treatment). Asterisks (*), and (***) denote statistical significance at *p* < 0.05, and *p* < 0.001 levels, respectively. Cells on the main diagonal represent a self-treatment comparison. The color intensity and hue reflect the magnitude and direction of differences in the *Sr* mean. The color scale ranges from −55.32 to 55.32% (panel (**a**)) and from −55.87% to 55.87% (panel (**b**)).

Clustering patterns based on the Tukey test after 6 h exposure (Figure 5a) showed that the highest *Sr* were observed in the biofilm and the control group (Cluster C4). Conversely, KCl (20 mM), KCl (30 mM), and GABA (10^−4^ M) (clusters C1 and C2) showed a significantly lower *Sr* with a mean difference of −55.17%, −45.61%, and −41.64%, respectively, compared to the control group. After 24 h exposure (Figure 5b), the control group and the biofilm maintained the highest *Sr*. Conversely, both KCl and GABA concentrations (clusters C′1, C′2, and C′3) showed significantly lower *Sr* compared to the control group (*p* < 0.0001), with mean differences ranging from −44.81% (GABA 10^−6^ M) to −55.71% (GABA 10^−4^ M) relative to the control.

### 3.2. Effect of Treatments on Spat Production (Pr)

**Figure 6:**
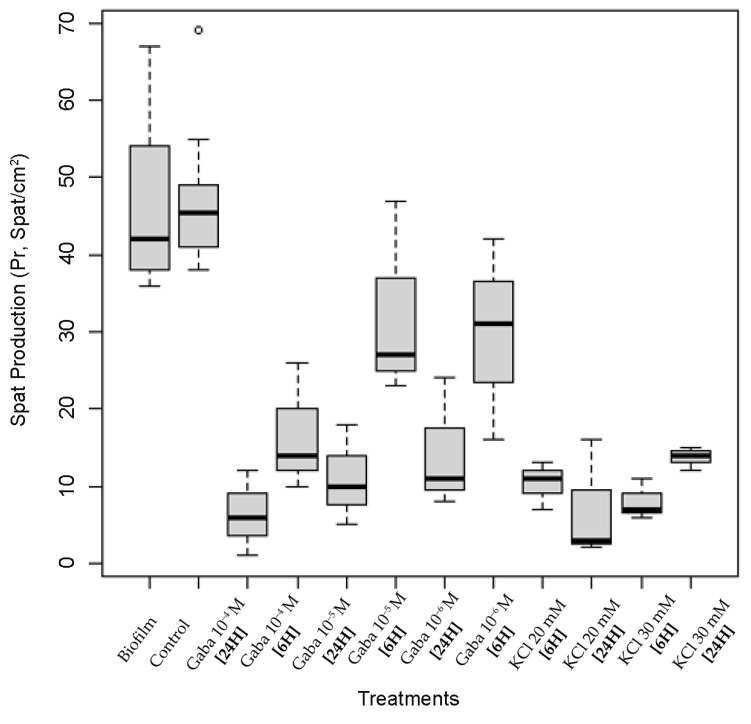
Spat production (*Pr*, spat/cm^2^) after 6 h and 24 h exposure to chemical treatments (GABA 10^−4^ M, GABA 10^−5^ M, GABA 10^−6^ M, KCl 20 mM, and KCl 30 mM), biofilm treatment, and control group. The open circle represents an outlier value.

Spat production (*Pr*, spat/cm^2^) was significantly higher in both the biofilm treatment (46.4 spat/cm^2^) and the control group (46.3 spat/cm^2^) than in the chemical treatments (Figure 6). Six hours of exposure time to GABA concentrations showed higher spat production than 24 h exposure. Larvae under KCl treatments showed very low spat production (6.9–13.8 spat/cm^2^), regardless of exposure time and concentration.

ANOVA results showed similar *Sr* and *Pr* between the biofilm treatment and the control group, and a negative effect of chemical treatments on both *Sr* (*F* = 80.7; *df*_num = 2; *df*_den = 18.7; *p* < 0.001) and *Pr* (*F* = 80.99; *df*_num = 2; df_den = 18.64; *p* < 0.001) (Table 2).

### 3.3. Effect of Treatments on Post-Larval Growth Rate (Gr)

**Figure 7:**
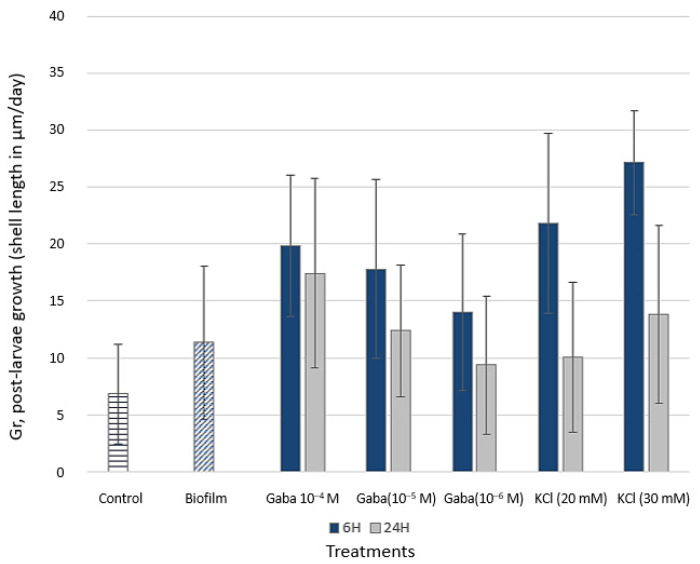
Post-larval growth rate (*Gr*, shell length in µm/day) after the first week in micro-nursery of pretreated larvae (GABA 10^−4^ M, GABA 10^−5^ M, GABA 10^−6^ M, KCl 20 mM, and KCl 30 mM) under two exposure times (6 h and 24 h).

The highest *Gr* after one week upon settlement was observed after 6 h exposure to both 30 mM KCl (27.2 µm/day) and 20 mM KCl (21.8 µm/day), and the lowest *Gr* (11.3 µm/day) was observed in the biofilm treatment and in the control group (Figure 7). Increasing initial GABA concentrations (from 10^−6^ M to 10^−4^ M) correlated with increasing *Gr* at both 6 h and 24 h exposure.

Exposure time of larvae to the chemical agents GABA and KCl negatively influenced post-larval *Gr* (*F* = 8.48; *df*_num = 1; *df*_den = 95.13; *p* < 0.01 and *F* = 41.33; *df*_num = 1; *df*_den = 60.11; *p* < 0.001, for GABA and KCl, respectively) (Table 3).

**Figure 8:**
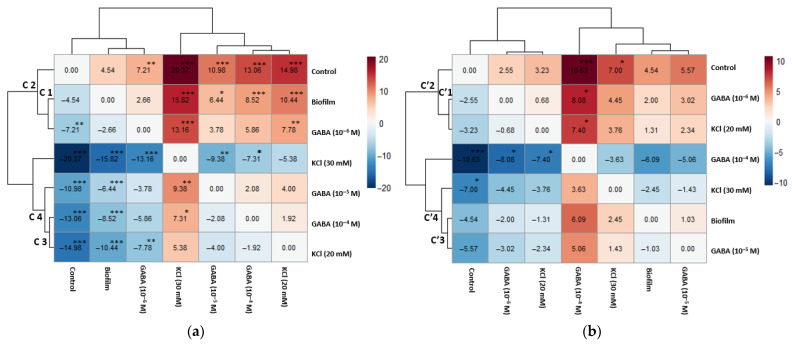
Heatmaps of Tukey HSD pairwise mean differences for post-larval *Gr* (shell length in µm/day) for biofilm, GABA, KCl, and control, (**a**) after 6 h exposure to chemical treatments (hierarchical dendrogram Cn), and (**b**) after 24 h exposure to chemical treatments (hierarchical dendrogram C′n). Upper diagonal cells contain the mean difference in *Gr* (µm/day) between the ‘Compared Treatment’ (column) and the ‘Reference Treatment’ (row). Lower diagonal cells show the difference between the Mean of Row Treatment and the Mean of Column Treatment. Asterisks (*), (**), and (***) denote statistical significance at *p* < 0.05, *p* < 0.01, and *p* < 0.001 levels, respectively. Cells on the main diagonal represent a comparison of a treatment to itself and are 0.00. The color intensity and hue reflect the magnitude and direction of differences in the *Gr* means. The color scale ranges from −20 to 20 µm/day (panel (**a**)) and from −10 to 10 µm/day (panel (**b**)).

Tukey’s test showed differences in post-larval *Gr* between treatments. At 6 h, KCl (30 mM) showed a significantly higher *Gr* than the control group (Figure 8a) (a mean difference of 20.37 µm/day (see Table 4)). At 6 h, GABA concentrations also showed a significantly higher *Gr* compared to the control, with a mean difference of 13.06 µm/day (GABA 10^−4^ M), 10.98 µm/day (GABA (10^−5^ M), and 7.21 µm/day (GABA (10^−6^ M). After 24 h (Figure 8b), KCl (30 mM) still showed higher *Gr* than the control, but its effect decreased to 7 µm/day. At 24 h, GABA (10^−4^ M) exhibited the highest mean difference relative to the control (10.63 µm/day).

Global ANOVA analyses showed that the treatment significantly influenced post-larval *Gr* (*F* = 28.97; *df*_num = 3; *df*_den = 71.77; *p* < 0.001) (Table 4).

### 3.4. Principal Component Analysis (PCA) and Redundancy Analysis (RDA) of Larvae Settlement (Sr), Post-Larval Growth (Gr), and Spat Production (Pr)

**Figure 9:**
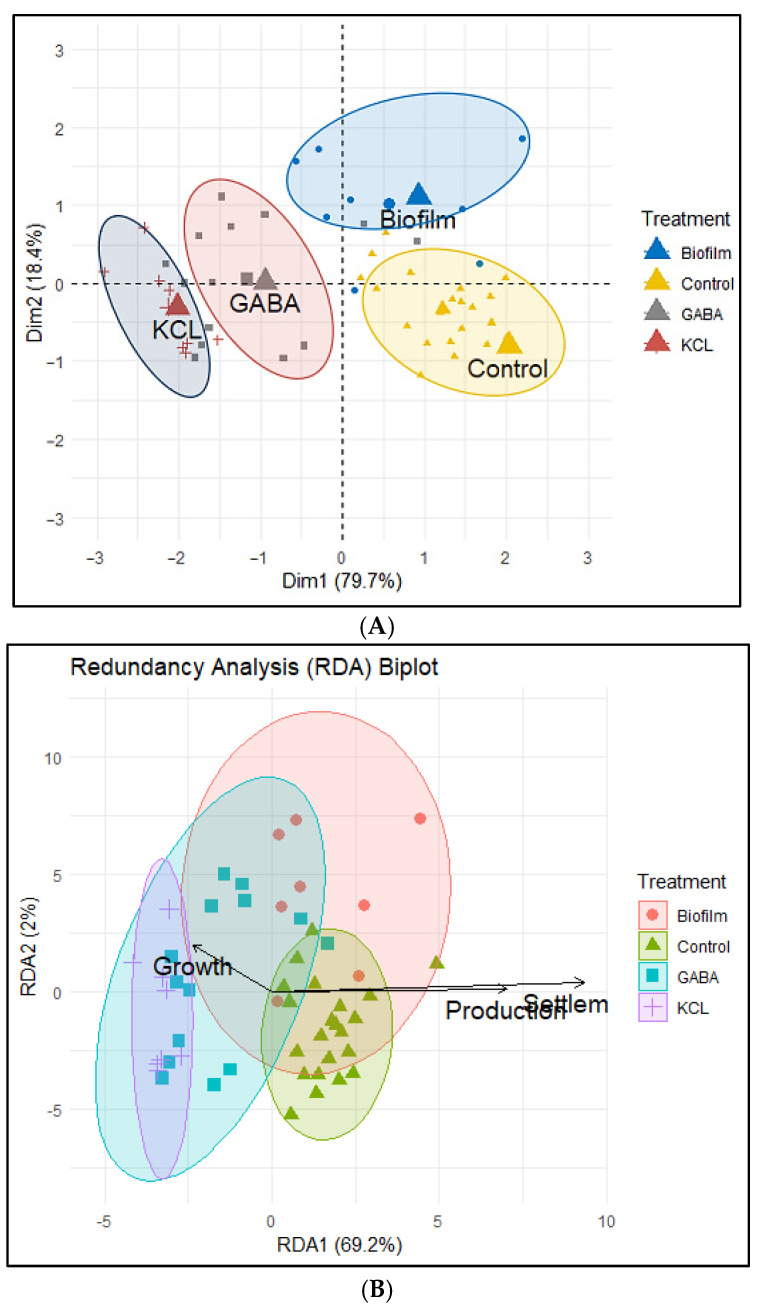
(**A**). Principal Component Analysis (PCA) of combinations of larvae settlement, spat production, and post-larval growth rate of *M. galloprovincialis*. Symbols represent individual data points for each treatment: blue circles (biofilm), yellow triangles (control), gray squares (GABA), and red plus signs (KCl). (**B**). Redundancy Analysis (RDA) of combinations of larvae settlement (*Sr*), spat production (*Pr*), and post-larval growth rate (*Gr*) in *M. galloprovincialis*. Symbols represent individual data points: red circles (Biofilm), green triangles (Control), cyan squares (GABA), and purple plus signs (KCl). Shaded ellipses represent the 95% confidence interval for each treatment.

The Principal Component Analysis (PCA) showed rough differences in *Sr*, *Pr*, and *Gr* between groups (control, biofilm, GABA, and KCl) (Figure 9A). The first dimension (Dim 1), which explains about 80% of the total variance, separates the chemical treatments (KCl and GABA) from the control and the biofilm treatment. This primary separation is driven by the fact that the chemical treatments resulted in higher post-larval growth (*Gr*), while the control and biofilm treatments were associated with higher settlement (*Sr*) and spat production (*Pr*). The remaining 18.4% of variance (Dim 2) establishes a main difference between the biofilm treatment and the control group.

Redundancy Analysis (RDA) biplot showed different responses of *Sr*, *Pr*, and *Gr* between groups (Figure 9B). The main driver of these differences (RDA1, explaining 69.2% of the variance) shows the control and biofilm groups associated with higher settlement rate (*Sr*) and spat production (*Pr*), while GABA and KCl treatments are linked to higher growth rate (*Gr*) in shell length.

## 4. Discussion

This study evaluated the effects of gamma-aminobutyric acid (GABA) and potassium chloride (KCl) as chemical inducers, alongside natural microalgal biofilm, on the settlement and metamorphosis of *M. galloprovincialis* pediveliger larvae in a nursery system.

### 4.1. Larvae Settlement

It is known that the thickness and complexity of biofilm generate high settlement efficiency in the pearl oyster (*Pinctada fucata*) [60,61,62] and in the Peruvian scallop (*Argopecten purpuratus*) [63,64]. Furthermore, diatom-dominated old biofilms significantly improved settlement in *Mytilus galloprovincialis* [65], *Mytilus edulis* [66], *Mytilus coruscus* [67], and *Aulacomya maoriana* [68]. The inductive property of these biofilms is attributed to the production of chemical cues, extracellular polymeric substances, and quorum-sensing signals [69]. Diatom-composed biofilms are considered very effective due to their high production of extracellular polymers (ECPs) [70] that mediate cell adhesion to surfaces and stabilize biofilms [71]. Consequently, larger amounts of ECPs have been positively correlated with metamorphosis and settlement induction in several species [72,73,74]. The similarity settlement between the control and biofilm treatments suggests that the effect of the intentional biofilm was masked. While the 48 h cleaning cycle presents a theoretical risk of secondary fouling, this was mitigated by the downwelling flow in the rearing system and confirmed by the visual lack of emergent biofilm on the control mesh. However, this mitigation must be considered alongside the operational context: the possibility of emergent secondary fouling on the control mesh cannot be fully ruled out as the system maintained continuous daily feeding and lacked mesh cleaning during the critical first week of settlement. The current lack of settlement enhancement in the biofilm treatment may, therefore, be due to the quality and maturity of the microalgal biofilm employed [60]. While the biofilm used in this study was sufficiently mature compared to previous studies on *M. galloprovincialis* [22], *Mytilus edulis* [66], and other marine invertebrates [70,75,76], it is possible that it was not old enough to have undergone critical microbial shifts or degradation of extracellular cues, resulting in a less effective biofilm relative to those studies reporting its positive effect on settlement. Another clue for the neutral role of the present biofilm is that it contained only one diatom strain (ranging 21–27% and mixed with two flagellate microalgae), as compared to multispecific diatom biofilms [77,78,79]. A third explanation points to biofilm eutrophication, potentially generated by nutrient availability alongside chemical inhibitors, which may have upregulated extracellular antibiotic resistance genes, eARGs [80], or microbial corrosion by impacting nitrogen and sulfur cycling [81], thus disrupting its productivity and masking the settlement effect.

In contrast to the biofilm, larval settlement was consistently low under the chemical treatments (GABA and KCl). This observation is consistent with previous research showing that while these compounds can induce metamorphosis, their effects are highly dependent on the type, concentration, and time of exposure. Therefore, our choice of 6 h and 24 h exposure times was driven by the need to assess not only immediate settlement induction but rather the sustained effect of GABA on larval survival and development. The lower settlement effectiveness of GABA observed, as compared to previously reported at higher concentration (10^−3^) during a shorter exposure time (30–60 min) [82], can be attributed to the extended exposure times (6 h and 24 h) applied. Although low concentrations (10^−4^–10^−5^) were tested, the prolonged exposure may induce sub-lethal stress or behavioral suppression (such as reduced ciliary beating) that masks the primary metamorphic induction cues. While these extended exposures are operationally convenient, this trade-off appears to result in reduced immediate settlement efficiency. In other instances, larvae of *Mytilus canaliculus*, *Mytilus edulis*, and *Crassostrea gigas* exhibited little to no response to GABA, emphasizing species-specific response variability [25,34,35,49,82,83,84,85]. The improved settlement rate of ~45% under lower GABA concentrations of this study, as compared to ~20% [82] at similar concentrations, emphasizes the importance of balancing exposure time and concentration to optimize settlement rates while minimizing the toxic effect of inducers. Larvae exposure to 20, 30, and 40 mM KCl produced optimal settlements (>90%) in *M. galloprovincialis* [82]. The efficiency of the potassium ion (K+) on settlement induction was also reported in various marine species [86,87,88,89,90,91]. Therefore, the lower rates of KCl-induced settlement observed in this study compared to previous ones may be attributed to a longer exposure time under the same KCl concentrations.

### 4.2. Post-Larval Growth Rate

The significant enhancement of post-larval development (13–17 µm/day) at moderate concentrations of GABA (10^−4^–10^−5^ M) under 6 h exposure supports previous observations in the mollusc *Clione limacine*, where GABA activated cerebral motoneurons involved in feeding behavior [92]. Namely, in marine bivalves, the nutritional absorption of amino acids positively affects metabolic efficiency, as has also been shown in *Mytilus* [93,94].

Bivalve larvae metamorphosis is an energy-intensive transitional phase characterized by physiological changes [95] that often result in stabilization or delay in larval growth. However, it was revealed that the application of biological and chemical treatments mitigated such growth delay. By the end of the current experimental phase 2 (before transfer to settlement), larvae exhibited a shell length growth rate of 10.66 µm/day. One week after the transfer, growth in the control group decreased as opposed to the treated groups (biofilm and chemical agents). Thus, the chemical treatments appear to have had an enhancing effect on post-larval growth rate. Notably, no studies have previously reported the positive influence of K+ ions on post-larval growth, as observed here at both 30 mM and 20 mM KCl (27.2 µm/day and 21.8 µm/day, respectively) at 6 h exposure. While the relationship between potassium ions (K+) and metabolic regulation has been previously reported in aquatic species, its role is controversial. For instance, K+ supplementation in shrimp larvae helps maintain proper ionic balance, enhances enzymatic activity, and supports energy production, allowing more energy to be allocated to growth and metabolic processes [96]. However, a concentration of 20 mM KCl did not show any effect on growth or feeding rates in the gastropod (*Crepidula fornicate*) juveniles [86].

### 4.3. Exposure Time

It is known that exposure time to chemical agents plays a major role in the success of settlement and development of larvae. Mechanistically, it has been suggested that the duration of exposure to GABA both enables its internalization by allowing binding to its receptors within neural pathways and interacts with external receptors to trigger metamorphosis [97]. Current results revealed that 6 h exposure time to GABA and KCl (at their highest concentrations assayed) had a negative effect on larval settlement but a positive effect on post-larval growth rate. However, this result is far from being generalizable to all species due to a positive increase in *Sr* previously reported in larvae of the pearl oyster *Pinctada fucata martensii*, i.e., in this species, an exposure of 6 h to 20 mM–50 mM KCl resulted in a settlement rate of 80–90% compared to the control group [46].

The low post-larval performance under 24 h treatment duration could be attributed to chemical toxicity or to undesired bacterial growth promoted by the chemicals [83]. Indeed, prolonged exposure to chemical inducers may cause ion transport dysregulation or osmotic stress, leading to cellular damage [98,99]. Furthermore, long-term chemical exposure may activate stress response pathways that ultimately inhibit metamorphosis and larval development [100]. These findings reveal a clear complexity of using chemical inducers in hatchery production. This study suggests that a short exposure (6 h) to GABA and KCl is sufficient to initiate the positive effects on settlement and growth, while a prolonged exposure could trigger negative physiological responses that inhibit development.

## 5. Conclusions

This study was conducted as an operational large-scale (mass production) trial to assess the role of chemical cues on larval settlement (*Sr*), post-larval growth (*Gr*), and spat production (*Pr*) of hatchery-produced *M. galloprovincialis*. Species-specific responses are a reasonable explanation for the different sensitivity of *M. galloprovincialis* larvae to GABA and KCl as compared to other bivalves [82]. Such contrasting outcomes may also be influenced by the physiological state of larvae, the exposure time, and subtle variations in the microbial environment. The biofilm-treated larvae showed a superior performance to the chemical agents in spat production. However, chemical treatments outperformed biofilm in post-larval growth rate. Based on this trade-off, maximizing long-term spat production is optimally achieved by utilizing natural settlement surfaces (biofilm), while aiming for a high post-larval growth rate requires short exposure (<6 h) to intermediate concentrations of GABA (10^−4^–10^−5^ M). Present results suggest to overtake the 70% settlement and 50 spat/cm^2^ in post-larval production previously reported [58], using improved multi-diatom biofilms rich in EPS in conjunction with short exposures (<6 h) to chemical inducers at the larvae stage to enhance settlement (*Sr*) and spat production (*Pr*).

## Figures and Tables

**Table 1:** One-factor ANOVA for the settlement rate (*Sr*, %) of larvae exposed to chemical treatments (GABA and KCl) after 6 h and 24 h exposure. *F*, Fisher test; *df*_num, numerator degrees of freedom; *df*_den, denominator degrees of freedom; ns, non-significant.

Treatment	Exposure Time	Mean *Sr* (±SD)	*df*_num	*df*_den	*F*	*p*-Value
GABA	6 h	36.57 ± 9.37	1	12.26	10.73	0.006
	24 h	14.93 ± 9.37				
KCl	6 h	14.48 ± 8.93	1	7.06	0.11	0.75 ^ns^
	24 h	13.15 ± 4.15				

**Table 2:** One-factor ANOVA for settlement rate (*Sr*, %) and spat production (*Pr*, spat/cm^2^) under biofilm and chemical treatments. *F*, Fisher test; *df*_num, numerator degrees of freedom; *df*_den, denominator degrees of freedom.

	Settlement Rate (*Sr*)					Production Rate (*Pr*)				
Treatment	Mean *Sr* (±SD) ^1^	*df*_num	*df*_den	*F*	*p*-Value	Mean *Pr* (±SD) ^1^	*df*_num	*df*_den	*F*	*p*-Value
Control	64.87 ± 9.75 ^b^	2	18.70	80.75	6.30 × 10^−10^	46.34 ± 6.97 ^b^	2	18.64	80.99	6.40 × 10^−10^
Biofilm	65.03 ± 15.16 ^b^					46.45 ± 15.15 ^b^				
Chemical	20.97 ± 15.27 ^a^					14.98 ± 6.97 ^a^				

^1^ A significant difference was observed between treatments with different superscripts (a,b).

**Table 3:** Results of ANOVA (one-factor) on post-larval growth rate (*Gr*) after chemical treatments (GABA and KCl) for 6 h and 24 h. *F* = Fisher test; *df*_num = numerator degrees of freedom; *df*_den = denominator degrees of freedom.

Treatment	Exposure Time	Mean *Gr* (±SD)	*df*_num	*df*_den	*F*	*p*-Value
GABA	6 h	17.27 ± 7.32	1	95.13	8.48	0.004
	24 h	13.09 ± 7.57				
KCl	6 h	23.91 ± 7.22	1	60.11	41.33	2.31 × 10^−8^
	24 h	12.05 ± 7.40				

**Table 4:** Global ANOVA (one-factor) for post-larval growth rate (*Gr*, shell length in µm/day) between treatments; *F* = Fisher test; *df*_num = numerator degrees of freedom; *df*_den = denominator degrees of freedom.

Treatment	Mean *Gr* (±SD) ^1^	*df*_num	*df*_den	*F*	*p*-Value
Control	6.80 ± 4.39 ^a^	3	71.77	28.97	2.20 × 10^−12^
Biofilm	11.35 ± 6.73 ^ab^				
GABA	15.54 ± 7.67 ^bc^				
KCl	18.26 ± 9.39 ^c^				

^1^ A significant difference was observed between treatments with different superscripts (a,b,c).

## Data Availability

All data generated are contained either in the article or are extendable upon request to the corresponding author.
